# Effects of Doped Elements (Si, Cr, W and Nb) on the Stability, Mechanical Properties and Electronic Structures of MoAlB Phase by the First-Principles Calculation

**DOI:** 10.3390/ma13194221

**Published:** 2020-09-23

**Authors:** Yongxin Jian, Zhifu Huang, Yu Wang, Jiandong Xing

**Affiliations:** 1 State Key Laboratory for Mechanical Behavior of Materials, School of Materials Science and Engineering, Xi’an Jiaotong University, Xi’an 710049, China; jiandongxing@xjtu.edu.cn; 2 Shaanxi Special Equipment Inspection and Testing Institute, Xi’an 710048, China; yuwang@stu.xjtu.edu.cn

**Keywords:** MoAlB phase, elemental doping, mechanical properties, electronic structures, first-principles calculations

## Abstract

First-principles calculations based on density functional theory (DFT) have been performed to explore the effects of Si, Cr, W, and Nb elements on the stability, mechanical properties, and electronic structures of MoAlB ternary boride. The five crystals, with the formulas of Mo_4_Al_4_B_4_, Mo_4_Al_3_SiB_4_, Mo_3_CrAl_4_B_4_, Mo_3_WAl_4_B_4_, and Mo_3_NbAl_4_B_4_, have been respectively established. All the calculated crystals are thermodynamically stable, according to the negative cohesive energy and formation enthalpy. By the calculation of elastic constants, the mechanical moduli and ductility evolutions of MoAlB with elemental doping can be further estimated, with the aid of B/G and Poisson’s ratios. Si and W doping cannot only enhance the Young’s modulus of MoAlB, but also improve the ductility to some degree. Simultaneously, the elastic moduli of MoAlB are supposed to become more isotropic after Si and W addition. However, Cr and Nb doping plays a negative role in ameliorating the mechanical properties. Through the analysis of electronic structures and chemical bonding, the evolutions of chemical bondings can be disclosed with the addition of dopant. The enhancement of B-B, Al/Si-B, and Al/Si-Mo bondings takes place after Si substitution, and W addition apparently intensifies the bonding with B and Al. In this case, the strengthening of chemical bonding after Si and W doping exactly accounts for the improvement of mechanical properties of MoAlB. Additionally, Si doping can also improve the Debye temperature and melting point of the MoAlB crystal. Overall, Si element is predicted to be the optimized dopant to ameliorate the mechanical properties of MoAlB.

## 1. Introduction

Binary transition metal borides are regarded as the superior candidates for high-temperature structural ceramics, due to their outstanding properties, such as high melting temperature, high hardness, and thermodynamic stability [1,2,3]. However, the inherent brittleness, poor oxidation resistance, as well as low damage tolerance have impeded the application of binary borides. To address these limitations, the researchers have tried to introduce an Al atomic layer into transition metal borides to form so-called MAB phases [4,5]. In MAB phases, single or double Al atomic layers are interleaved into a transition metal boride sublattice. The structures of MAB phases are analogous to the previous MAX phases, which show significant advantages for both metal and ceramics with good heat and electricity conduction, and considerable ductility and damage tolerance [6,7,8,9,10]. In this case, MAB phases are also expected to possess the excellent properties of both metals and borides.

Jeitschko et al. [4] first discovered MAlB (space group Cmmm) ternary transition metal borides in 1966. Subsequently, extensive research has focused on the synthesis of single crystal ternary borides, and determining their crystal structures [11]. In 2016, Kota et al. [5,12] synthesized the polycrystalline MoAlB bulk by a hot-pressing method, and investigated its oxidation resistance, heat and electrical conductivity, thermal expansion, and mechanical properties. Their results suggest the significant potential of MoAlB ceramic in application to high-temperature fields. Afterwards, researchers all over the world conducted extensive investigations on the synthesized methods, mechanical properties, thermal shock behaviors, oxidation behaviors, as well as the friction and wear behaviors of MoAlB ceramics [12,13,14,15,16,17,18,19,20,21,22,23,24]. As reported, MoAlB exhibited excellent high-temperature oxidation resistance and compressive properties, but the fracture toughness was still insufficient, especially considering its potential application as structural parts. In spite of the introduction of Al atomic layer, the MoAlB phase still fails in its brittleness, by way of cleavage fractures. In this case, addressing the problem of the intrinsic brittleness of MoAlB is of great significance for its industrial application.

Traditionally, the elemental doping method is usually executed to ameliorate the mechanical properties of metallic materials as well as intermetallics (for instance carbides and borides) [25,26,27,28,29,30]. Jian et al. [29,31] improved the fracture toughness of Fe_2_B without sacrificing the hardness by appropriate Cr or Mn addition. Shen et al. [32] managed to enhance the hardness and flexure strength of Mo_2_FeB_2_ ceramic with Cr dopant. For MoAlB, Shigeru et al. [33] tried to investigate the effects of the transition metal atoms (Cr, W, V, and Nb et al.) on the hardness and oxidation resistance of MoAlB. In their study, only (Mo_x_Cr_1−x_)AlB and (Mo_x_W_1−x_)AlB were successfully obtained by the flux method using molten aluminum as the solvent, and W was found to play a positive role in improving the hardness. However, the intrinsic reason for the mechanical evolution originating from elemental doping was not clarified deeply. Recently, Ma et al. [34] found that Si addition can facilitate the formation of MoAl_1−x_Si_x_B so as to improve the strength and hardness of MoAlB. Similarly, the strengthening mechanism by Si doping was substantially not discussed in their work. Thus, the affecting mechanism of elemental doping on MoAlB phase urgently needs to be clarified, which can also help to find new strategies to ameliorate the mechanical properties, especially for the toughness. 

In recent years, first-principles calculation based on density functional theory (DFT) has shown considerable accuracy in predicting the phase stability, lattice parameters, mechanical properties, and electronic structures of compounds [35,36,37,38]. Thus, theoretical calculation has developed to be an effective tool to predict and design new compounds with extraordinary properties. Simultaneously, the abundant information from calculations can help to disclose the intrinsic reason, which cannot be observed through experimental methods. In this case, this work mainly focuses on systematically investigating the effects of Si, Cr, W, and Nb doping on the stability, elastic, and physical properties of MoAlB. By analyzing the evolutions of the electronic structures and bonding characters, the affecting mechanism has been discussed comprehensively. In this case, this work is expected to provide guideline for improving the mechanical properties of MoAlB ceramic, by way of element doping. 

## 2. Methodology 

In this work, the calculations were performed by using density functional theory (DFT) implemented in the Cambridge Serial Total Energy Package (CASTEP) code [39,40]. The exchange correlation energy was treated according to the generalized gradient approximation (GGA) in the scheme of Perdew–Burke–Ernzerhof (PBE) [41,42]. Ultrasoft pseudopotentials were employed to describe the interactions between the ionic core and valence electrons in the reciprocal space [43]. The plane wave energy cutoff was fixed at 600 eV. Brillouin zone sampling was conducted by Monkhorst–Pack method, with the special k-point mesh of 10 × 10 × 10. The crystal was fully optimized by modifying the lattice parameters and internal atomic positions using the Broyden–Fletcher–Goldfarb–Shannon (BFGS) minimization scheme [44]. The total energy of the lattice structure was minimized to converge to 5 × 10^−6^ eV/atom, along with the ionic Hellmann–Feynman force components, ionic displacements, and stress tensor components being converged to within 0.01 eV/Å, 5 × 10^−4^ Å, and 0.02 GPa, respectively.

**Figure 1:**
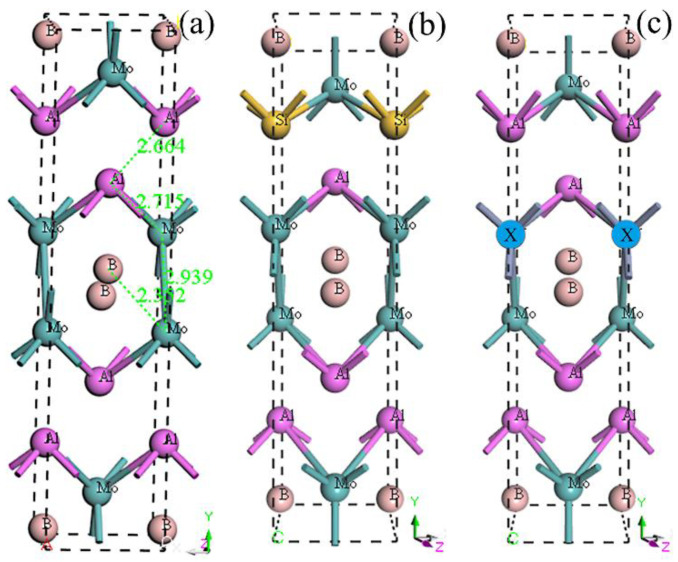
Crystal structures of MoAlB: (**a**) Initial MoAlB (bond distance unit: Å); (**b**) Si doped MoAlB; (**c**) X doped MoAlB.

Figure 1a illustrates the crystal cell of initial MoAlB, with an orthorhombic lattice structure. Mo, Al, and B atoms occupy the 4c Wyckoff positions of the *Cmcm* space group [11]. In this research, the crystal model of Si doped MoAlB was established by replacing one Al atom by Si, considering the similar atomic radius, as shown in Figure 1b. However, given the similarity among Cr, W, Nb, and Mo, the crystal structure of X (X representing Cr, W, or Nb) doped MoAlB was obtained with one X atom occupying the initial Mo position [45]. The crystal structure of X doped MoAlB is shown in Figure 1c. In this case, five crystals with the formulas of Mo_4_Al_4_B_4_, Mo_4_Al_3_SiB_4_, Mo_3_CrAl_4_B_4_, Mo_3_WAl_4_B_4_, and Mo_3_NbAl_4_B_4_ were investigated in this work, involving the stability, mechanical properties, electronic structures, and thermal properties.

## 3. Results and Discussion

### 3.1. Equilibrium Structure and Stability

After full optimization, the calculated lattice parameters of the five crystal structures are shown in Table 1. Compared with experimental results, the theoretical results of MoAlB in this work show great agreement, with the deviations less than 0.56% [5,46]. In addition, the results in this work are highly consistent with the reported theoretical results calculated with the same method [47]. Thus, the calculating method and parameters implemented in this work are supposed to be suitable, which ensures highly accurate predictions of other doped crystals. From Table 1, it can be found that the volume of crystal cell shrunk after adding Si and Cr, while it tended to swell with the addition of W and Nb. This phenomenon can be easily understood considering the deviations between the doped atom and the initial one. In addition, Cr doped MoAlB crystal shows the lowest density, which decreases by 5.7% compared with the non-doping MoAlB. On the contrary, W doping dramatically increases the density of MoAlB by about 16.4%.

Thermostability of the crystal is an important parameter in evaluating the possibility of the doping reaction, and can be estimated by the parameters of cohesive energy (*E_coh_*) and formation enthalpy (∆*H_r_*). In general, negative values of *E_coh_* and ∆*H_r_* indicate the crystal structure to be thermodynamically stable. The *E_coh_* and ∆*H_r_* of a compound can be calculated according to Equations (1) and (2) [48,49,50,51]:

(1) $$E_{coh}(A_{x}B_{y}C_{z}D_{m})=\frac{E_{tol}(A_{x}B_{y}C_{z}D_{m})-xE_{iso}(A)-yE_{iso}(B)-zE_{iso}(C)-mE_{iso}(D)}{x+y+z+m}$$

(2) $$\Delta H_{r}(A_{x}B_{y}C_{z}D_{m})=\frac{E_{tol}(A_{x}B_{y}C_{z}D_{m})-xE_{bulk}(A)-yE_{bulk}(B)-zE_{bulk}(C)-mE_{bulk}(D)}{x+y+z+m}$$

where *E_tol_* represents for total energy; *E_iso_* and *E_bulk_* represent the energy of an isolated atom and a single atom in bulk state, respectively. From Table 1, it can be concluded that the all five calculated crystal structures are thermodynamically stable in view of the negative values of *E_coh_* and ∆*H_r_*. From the perspective of *E_coh_*, Si doped MoAlB is the most stable crystal due to its smallest *E_coh_*, and the stability of crystal structures can be ranked in the order, Mo_4_Al_3_SiB_4_ > Mo_3_WAl_4_B_4_ > Mo_4_Al_4_B_4_ > Mo_3_NbAl_4_B_4_ > Mo_3_CrAl_4_B_4_. On the other hand, the negative formation enthalpies also prove all the crystals structures to be thermodynamically stable. In this context, it can be concluded that the doping of Si, Cr, W, and Nb should be realized in experiments.

### 3.2. Elastic Properties

The mechanical properties can be investigated by calculating the elastic constants of the crystals. In this work, the elastic constants have been evaluated by the stress-strain method, where the stress-strain curve was described by generalized Hooker’s law, as shown in Equation (3):

(3) $$\sigma_{ij}=C_{ijkl}\varepsilon_{ij}$$

where *σ_ij_* represents for the stress tensor; *εij* and *Cijkl* represent the Lagrangian strain tensor and elastic constant tensor, respectively. The elastic constants (*C_ij_*) of all the five crystals have been calculated and listed in Table 2. The second order elastic constants of MoAlB show pretty good agreement with the reported results [37,47], indicating the reliability of our calculation. The mechanical stability of compounds can be justified according to Boron–Huang’s criteria [52,53,54]. For the MoAlB crystals with orthorhombic lattice, the general criteria can be expressed as Equation (4):

(4) $$\begin{array}{l} C_{11}C_{12}-C_{21}{}^{2}>0;C_{22}C_{33}-C_{23}{}^{2}>0;C_{33}C_{11}-C_{31}{}^{2}>0;\\ C_{11}C_{22}C_{33}+2C_{12}C_{23}C_{31}-C_{11}C_{23}{}^{2}-C_{22}C_{31}{}^{2}-C_{33}C_{12}{}^{2}>0;\\ C_{ij}>0\left(i=j=1∼6\right) \end{array}$$

According to the constants in Table 2, all five crystal structures are mechanically stable, as they fully satisfy the above stability criterion. Like the MAX phases, the *C*_11_, *C*_22_, and *C*_33_ of MoAlB crystal, representing elastic stiffness against principal strain, are much higher than those against shear strains (*C*_44_, *C*_55_ and *C*_66_). In other words, MoAlB crystal is considerably incompressible along *a*, *b*, and *c* crystal axis, but shows relatively weaker resistance to the shear deformation on the (1 0 0), (0 1 0) and (0 0 1) planes. Furthermore, it can be observed that *C*_11_ and *C*_33_ are higher than *C*_22_ for all five crystals, indicating that MoAlB is more incompressible along the *a* and *c* axes [38]. The weaker incompressibility along the *b* axis may be attributed to the double-layer Al. *C*_33_ is the largest among all the elastic constants, which signifies the strongest covalent bonding along the *c* axis. Compared with initial MoAlB, Si and W doping are beneficial to improving the incompressibility along the *a* and *c* axes, with the increase of *C*_11_ and *C*_33_. On the other hand, it is apparent that the resistance to shear on the (0 1 0) plane is the weakest considering the smallest *C*_55_ value. However, the (1 0 0) plane is expected to show the strongest resistance to shear. Intrinsically, the elastic properties are mainly responding to the chemical bonding in the crystal, which will be deeply discussed thereinafter.

The ductility and brittleness of a compound can be verified by the value of Cauchy pressure on some extent [38]. If the Cauchy pressure is negative, the compound is expected to be brittle in nature. According to Table 2, Cauchy pressure of each calculated crystal can be calculated by the equation of (*C*_12_*–C*_44_). It can be deduced from the negative Cauchy pressures that the calculated MoAlB crystals are intrinsically brittle. Furthermore, the ductility relation is Mo_4_Al_3_SiB_4_ > Mo_3_NbAl_4_B_4_ > Mo_3_WAl_4_B_4_ > Mo_3_CrAl_4_B_4_ > Mo_4_Al_4_B_4_, by comparing the values of Cauchy pressure. In this context, it can be concluded that the addition of Si, Nb, W, and Cr benefits the improved ductility of MoAlB.

The bulk modulus (*B*), shear modulus(*G*), and Young’s modulus(*E*) can be estimated based on the elastic constants (*C_ij_*), according to the Voigt–Reuss–Hill (VRH) scheme [55]. The VRH approximation means the average of Voigt and Reuss approximations. Herein, Voigt approximation, based on the assumption of uniform strain, decides the upper bounds of the elastic modulus, as expressed by Equations (5) and (6):

(5) $$B_{V}=\frac{1}{9}(C_{11}+C_{22}+C_{33})+\frac{2}{9}(C_{12}+C_{23}+C_{13})$$

(6) $$G_{V}=\frac{1}{15}(C_{11}+C_{22}+C_{33})-\frac{1}{15}(C_{12}+C_{23}+C_{13})+\frac{1}{5}(C_{44}+C_{55}+C_{66})$$

And Reuss approximation, based on the assumption of uniform stress, decides the lower bounds. It can be defined as the elastic compliances (*S_ij_*) which are the inverse matrix of *C_ij_*, as expressed by Equations (7) and (8):

(7) $$\frac{1}{B_{R}}=(S_{11}+S_{22}+S_{33})+2(S_{12}+S_{23}+S_{13})$$

(8) $$\frac{1}{G_{R}}=\frac{4}{15}(S_{11}+S_{22}+S_{33})-\frac{4}{15}(S_{12}+S_{23}+S_{13})+\frac{1}{5}(S_{44}+S_{55}+S_{66})$$

Finally, the *B*, *G*, and *E* can be obtained following Equations (9)–(11), and Poisson’s ratio (*ν*) can be calculated according to Equation (12).

(9) $$B=\frac{1}{2}(B_{V}+B_{R})$$

(10) $$G=\frac{1}{2}(G_{V}+G_{R})$$

(11) $$E=\frac{9BG}{3B+G}$$

(12) $$\nu =\frac{3B-2G}{2(3B+G)}$$

**Figure 2:**
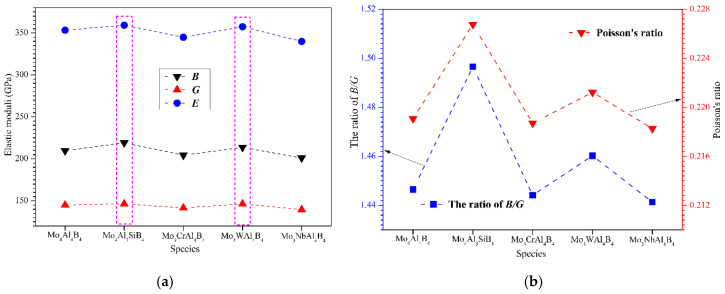
Mechanical properties of MoAlB crystals: (**a**) elastic moduli; (**b**) *B*/*G* and Poisson’s ratios.

Table 3 lists the calculated bulk moduli (*B*), shear moduli (*G*), and Young’s moduli (*E*) of all five calculated crystals. Additionally, the moduli data at 300K, measured by high-temperature resonant ultrasound spectroscopy (RUS) by Kota et al., have been provided as a comparison [56]. As shown, the calculated *E* of MoAlB is a little underestimated in this work compared with the experimental result. The calculated bulk modulus, shear modulus, and Young’s modulus have been plotted in Figure 2a. First, it can be found that Young’s moduli of MoAlB are considerably higher, with the values larger than 350 GPa indicating the strong chemical bonding inside the crystal. From Figure 2a, it can be visibly observed that the bulk modulus (*B*) is substantially larger than the shear modulus (*G*). Intriguingly, Si and W doped MoAlB exhibits a relatively higher elastic moduli compared to the others. This implies Si and W doping can help enhance the bonding interactions among the atoms. However, Cr and Nb doping seem to be detrimental to the elastic moduli. On the other hand, Pugh’s ratio can be obtained by calculating the ratio of *B/G*. *B/G* ratio is usually used to judge the ductile and brittle character of a solid [57]. A higher *B/G* value indicates a higher ductility, with a critical value of 1.75. Thus, the five calculated MoAlB crystals can be classified as brittle materials, with a *B/G* value less than 1.75, which is totally consistent with the prediction from Cauchy pressure. However, according to the previously reported experimental results [5,14,17,22], MoAlB exhibits considerable toughness and damage tolerance. In this case, the predication about the toughness of MoAlB may not be so dependent as other compounds on the value the of B/G ratio. This phenomenon agrees well with the literature’s reports that Pugh’s ratio may not necessarily be a strong indication of ductility, for the ternary layered compounds, such as MAB and MAX phases [36,58,59], because the effects of the layered structure, and significantly weak bonds of Al, on ductility, are underestimated during the calculation. Figure 2b reveals the Poisson’s and *B*/*G* ratios of the calculated MoAlB crystals. As shown, Mo_4_Al_3_SiB_4_ crystal possesses the highest *B*/*G* and Poisson’s ratios, indicating superior ductility. By comparison, it can be concluded that Si and W doping can play a positive role in improving the ductility of MoAlB, while Cr and Nb doping seem to make adverse contributions. 

Poisson’s ratio is also one of the important parameters in assessing the solid’s ductility and brittleness. With the critical value of 0.26, the material with a Poisson’s ratio lower than this usually behaves in a brittle manner, otherwise the material is expected to be ductile. In this context, the five calculated MoAlB crystals are all classified as brittle materials. This shows great consistency with the results of Cauchy’s pressure and *B/G* ratio. Moreover, the variation of Poisson’s ratio is also in good agreement with the B/G ratio. In summary, Si and W doping can enhance the elastic moduli and improve the ductility of MoAlB, which is expected to be an effective method to optimize the mechanical properties.

### 3.3. Elastic Anisotropy

The anisotropy of mechanical properties is a key factor that affects the material’s properties. Microcracks usually appear, originating from the mechanical anisotropy of materials. In MoAlB crystal, the elastic modulus along different principal axes are expected to differ from each other, considering the difference in the elastic constants in Table 2. To reveal the anisotropic features, the universal anisotropic index (*A^U^*), percent anisotropies (*A^B^* and *A^G^*), and shear anisotropic indexes (*A*_1_, *A*_2_ and *A*_3_) have been calculated in this work according to Equations (13)–(18):

(13) $$A^{U}=5\frac{G_{V}}{G_{R}}+\frac{B_{V}}{B_{R}}-6\geq 0$$

(14) $$A_{B}=\frac{B_{V}-B_{R}}{B_{V}+B_{R}}$$

(15) $$A_{G}=\frac{G_{V}-G_{R}}{G_{V}+G_{R}}$$

(16) $$A_{1}=\frac{4C_{44}}{C_{11}+C_{33}-2C_{13}}\;\mathrm{for}\;(100)\;\mathrm{plane}$$

(17) $$A_{2}=\frac{4C_{55}}{C_{22}+C_{33}-2C_{23}}\;\mathrm{for}\;(010)\;\mathrm{plane}$$

(18) $$A_{3}=\frac{4C_{66}}{C_{11}+C_{22}-2C_{12}}\;\mathrm{for}\;(001)\;\mathrm{plane}\;$$

Table 4 lists the calculated anisotropic indexes of MoAlB crystals, with and without elemental doping. First, it can be found that Mo_4_Al_3_SiB_4_ and Mo_3_WAl_4_B_4_ have relatively smaller *A^U^*, compared with the initial MoAlB, implying that the crystals tend to be more isotropic after Si and W doping. In addition, the *A_B_* and *A_G_* show the same varying trend with Si and W addition. Generally, directional covalent bonding plays a significant role in affecting the crystal’s anisotropy, while the metallic bonding contributes to improving the isotropy. Thus, it can be hypothesized that Si and W doping can help ameliorate the metallic character of MoAlB, which is beneficial to the crystal’s ductility. This results is in good agreement with the Pugh’s and Poisson’s ratios discussed above. Then, *A*_1_, *A*_2_, and *A*_3_ reveal the anisotropies of shear moduli on the (1 0 0), (0 1 0), and (0 0 1) planes, respectively. Mo_3_CrAl_4_B_4_ shows the smallest *A*_1_ and Mo_4_Al_3_SiB_4_ shows the smallest *A*_3_, while Mo_3_WAl_4_B_4_ and Mo_4_Al_4_B_4_ share the smallest *A*_2_. In other words, Mo_3_CrAl_4_B_4_, Mo_4_Al_3_SiB_4_, Mo_3_WAl_4_B_4_, and Mo_4_Al_4_B_4_ have the most isotropic shear moduli on the (1 0 0), (0 0 1), and (0 1 0) planes, respectively. Overall, Mo_3_NbAl_4_B_4_ is regarded as the most anisotropic crystal due to the relatively high anisotropic index. 

**Figure 3:**
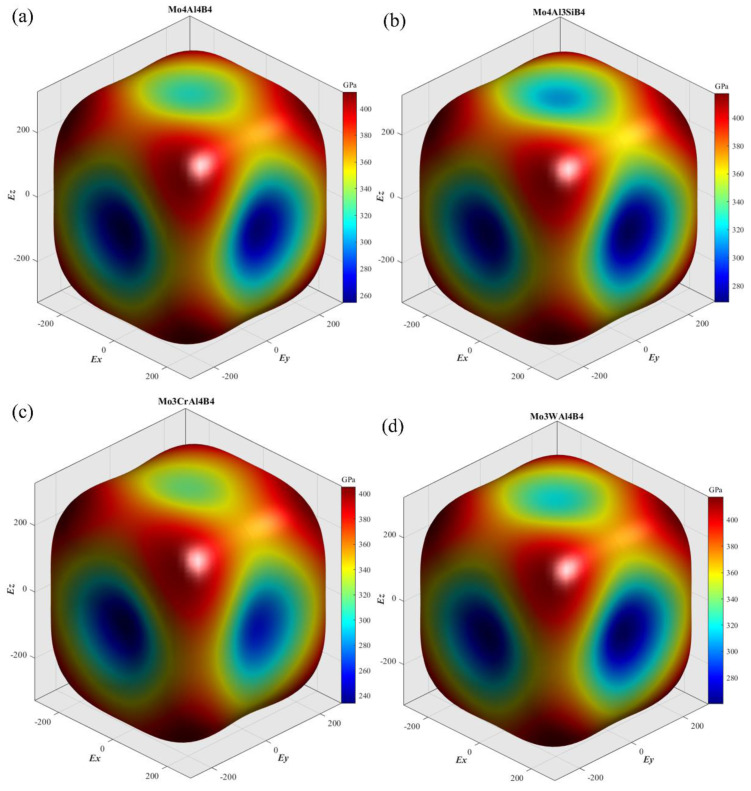

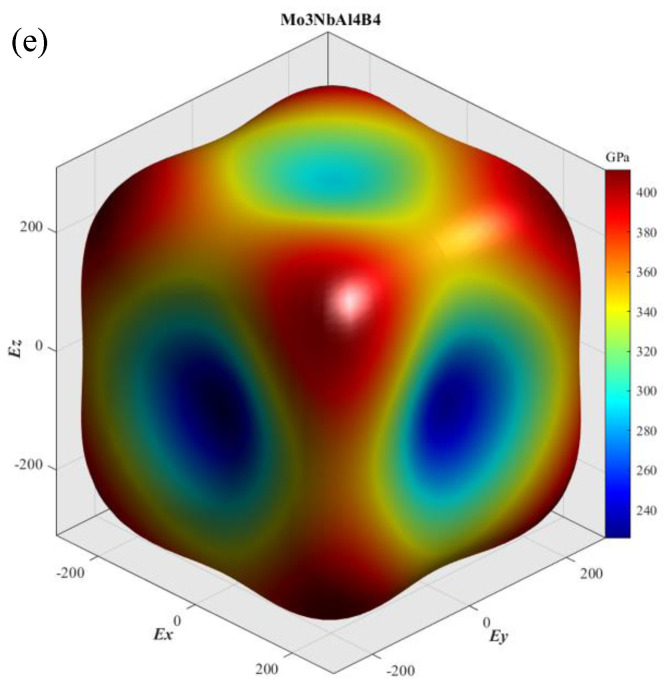
The spatial surface contours of Young’s modulus: (**a**) Mo_4_Al_4_B_4_; (**b**) Mo_4_Al_3_SiB_4_; (**c**) Mo_3_CrAl_4_B_4_; (**d**) Mo_3_WAl_4_B_4_; (**e**) Mo_3_NbAl_4_B_4_.

**Figure 4:**
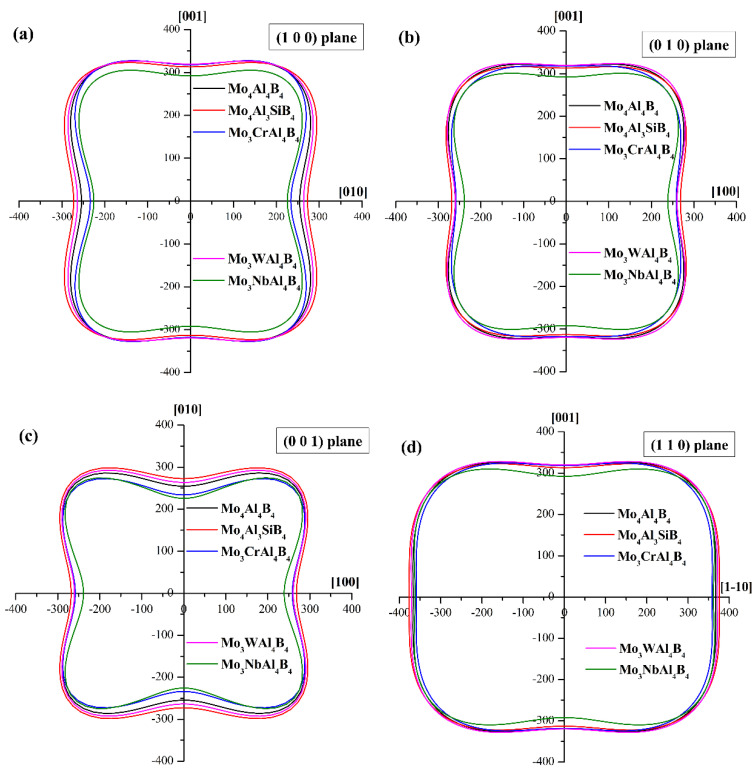
Planar projections of the Young’s modulus (GPa) on different planes: (**a**) on the (1 0 0) plane; (**b**) on the (0 1 0) plane; (**c**) on the (0 0 1) plane; (**d**) on the (1 1 0) plane.

Young’s modulus is an important parameter to evaluate the mechanical properties of a material. Thus, the anisotropic features of Young’s modulus are worthwhile to be clearly elucidated. The spatial surface contours are practical and visible, giving insight to the anisotropies of Young’s modulus [37]. In this case, the spatial surface contours of Young’s moduli, as a function of direction, have been depicted according to Equation (19):

(19) $$\frac{1}{E}=l_{1}{}^{4}S_{11}+l_{2}{}^{4}S_{22}+l_{3}{}^{4}S_{33}+2l_{1}{}^{2}l_{2}{}^{2}S_{12}+2l_{1}{}^{2}l_{3}{}^{2}S_{13}+2l_{2}{}^{2}l_{3}{}^{2}S_{23}+l_{2}{}^{2}l_{3}{}^{2}S_{44}+l_{1}{}^{2}l_{3}{}^{2}S_{55}+l_{1}{}^{2}l_{2}{}^{2}S_{66}$$

where *l*_1_, *l*_2_, and *l*_3_ are the directional cosines; *S_ij_* represents the elastic compliance constant. In spherical coordinates: *l*_1_ = sin *θ* cos *ψ*, *l*_2_ = sin*θ* sin *ψ* and *l*_3_ = cos *θ*, respectively. Figure 3 exhibits the three-dimensional surface contours of Young’s modulus of the five calculated crystals. As seen, the Young’s modulus of MoAlB shows great anisotropy, because the isotropic crystal usually has a spherical contour shape. The Young’s modulus of the five calculated crystals is the largest along the [1 1 1] crystal direction. On the contrary, the Young’s modulus seems to be the smallest along the [0 1 0] crystal direction. Overall, the contour shapes of the five crystals have considerable similarity, which may be attributed to the small difference in their lattice structures. To clarify the differences on different feature crystallographic planes, the planar projections on the (1 0 0), (0 1 0), (0 0 1), and (1 1 0) planes have been depicted in Figure 4. It can be noticed that the Young’s moduli on the (1 0 0) and (0 1 0) planes are obviously more anisotropic than those on the other two planes. Compared with the initial MoAlB crystal, the Mo_4_Al_3_SiB_4_ and Mo_3_WAl_4_B_4_ show more isotropic Young’s modulus distribution, while the distributions of Mo_3_CrAl_4_B_4_ and Mo_3_NbAl_4_B_4_ are more anisotropic. This result agrees well with the discussion on the anisotropic parameter, *A^U^*. On the other hand, it can be observed that, for five calculated crystals, the Young’s moduli along the [1 1 1] direction are the largest, while those along [0 1 0] are the smallest. Along the [1 1 1] direction, the Young’s modulus is higher than 400GPa, indicating the superior stiffness. As for the three principal axes, the relationship of Young’s modulus is *E*_[0 0 1]_ > *E*_[1 0 0]_ > *E*_[0 1 0]_, which is consistent with the spatial contours in Figure 3. Furthermore, it can be found that Si and W doping can account for the enhancement of Young’s modulus, especially along the [0 1 0] direction. The Young’s modulus along [0 1 0] can be improved from 254.60 to 272.74 and 263.68 GPa after Si and W doping, respectively. In general, the low Young’s modulus relates tightly with poor energy. Thus, Si and W doping are supposed to improve the uniaxial tension resistance along [0 1 0] of MoAlB.

### 3.4. Electronic Structures

**Figure 5:**
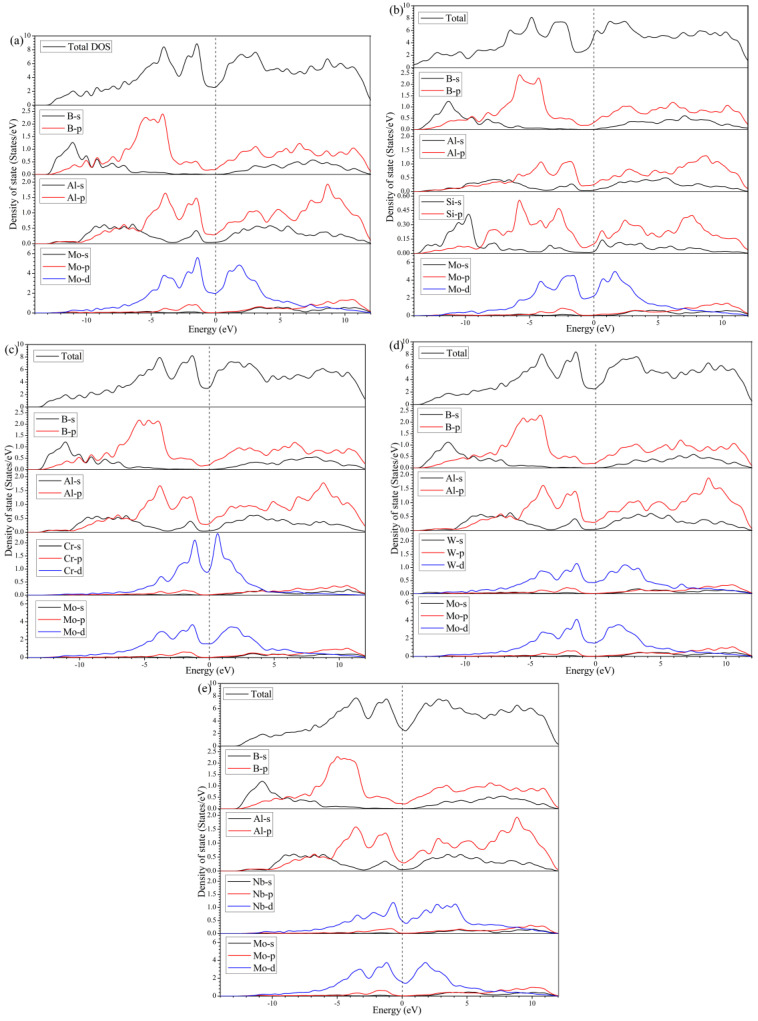
The total, and partial, density of states of: (**a**) Mo_4_Al_4_B_4_; (**b**) Mo_4_Al_3_SiB_4_; (**c**) Mo_3_CrAl_4_B_4_; (**d**) Mo_3_WAl_4_B_4_; (**e**) Mo_3_NbAl_4_B_4_.

To further put insight into the bonding character of MoAlB, as well as the elementally doped crystals, the electronic structures were explored, including the density of states (DOS) and electron density distribution. Figure 5 illustrates the total density of states (TDOS) and the partial density of states (PDOS) of the five calculated crystals. For all five crystals, the values of the DOS, at Fermi energy level (*E_f_*), are larger than zero, implying the metallic character of MoAlB. In addition, it is noticed that the *E_f_* exactly locates in the valley position of the TDOS, which is a portent of decent stability. By analyzing the PDOS of different atoms, it can be found that the DOS at *E_f_* is mainly contributed to by the *d* states of Mo or the corresponding metal atoms. In other words, Mo, or the doped transition metal atoms, play the dominant role in the electronic conduction of MoAlB. 

From Figure 5a, the valence band below −7.0 eV is mainly constituted from the *s* and *p* states of B and Al. The corresponding hybridizations account for the chemical bonding between B-B, Al-B, and probably Al-Al in MoAlB. From −7.0 to −3.0 eV, the highest peak is mainly attributed to the strong hybridizations among B-*s*, Al-*p,* and Mo-*d* orbitals, which renders into the formations of strong covalent bonding of Mo-B and Al-B. Moreover, the considerably high valence bands, with the peak centered around −1.2 eV, are mainly composed of the hybridization between Al-*p* and Mo-*d* states, indicating the relatively weak Mo-Al bonds. In Si doped MoAlB, the *s* and *p* states of Si make significant contributions to the valence bands below −7.0 eV, as shown in Figure 5b, which is possibly to facilitate the formation of Si-B bonds. In addition, the valence bands from −7.0 to −3.0 eV mainly originate from B-*p*, Al-*p*, Si-*p,* and Mo-*d* states, indicating the formation of covalent bonds between Mo, Al, B, and Si atoms, except for the initial Mo-B and Al-B bonds. Thus, the elastic properties of MoAlB are supposed to be improved due to the enhancement of internal bonding. However, after the doping of transition metal atoms of Cr, W, or Nb, the main differences in the states hybridization can be found to appear from −7.0 eV to *Ef,* originating from the contribution of their *d* states, as shown in Figure 5c to e. Herein, W is found to show the strongest hybridization with Mo, Al, and B, at around −4.0 eV, where the DOS peak is located, as shown in Figure 5d. In this case, the chemical bondings of W with Mo, Al, and B are hypothesized to be consequently intensified. Nevertheless, Cr and Nb mainly participate in the hybridization with Mo and Al at around −1.2 eV, which is deemed to affect the bonds of Mo (Cr or Nb)-Al. 

**Figure 6:**
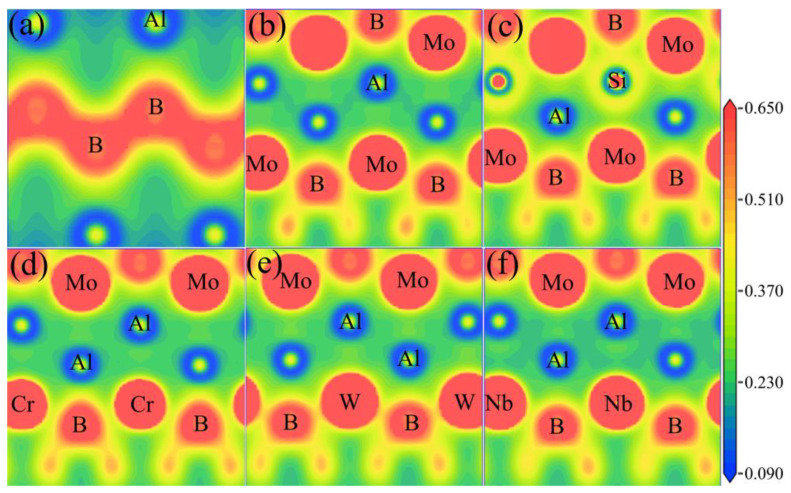
Electron density of the (1 0 0) plane of Mo_4_Al_4_B_4_ (**a**), and the (1 0 1) plane of (**b**) Mo_4_Al_4_B_4_, (**c**) Mo_4_Al_3_SiB_4_, (**d**) Mo_3_CrAl_4_B_4_, (**e**) Mo_3_WAl_4_B_4_, (**f**) Mo_3_NbAl_4_B_4_.

To clearly elucidate the type of chemical bonding as well as the corresponding evolution of the bonding strength with elemental doping, the bond lengths and overlapped populations have been analyzed and listed in Table 5. Simultaneously, the electron density distributions on the (1 0 0) and (1 0 1) crystal planes have been depicted as shown in Figure 6. From Table 5, it can be visibly found that the B-B bond has the shortest bond length and largest population (much larger than 1), indicating the strongest B-B covalent bonding. This conclusion can be confirmed from Figure 6a, in which a significantly high electron density can be observed between B-B atoms. The bonding between B-B shows apparently directional characters. In addition, Al-B and Mo-B bonds have overlapped populations much smaller than one, indicating mixed ionic and covalent bondings. However, the populations of Al-Al and Al-Mo bonds are extremely close to one, implying a significant tendency to covalent bonding. Herein, a negative value of overlapped population appears, basically for the bonds with a long distance. Generally, the negative population implies the antibonding or nonbonding between the two atoms.

After the introduction of Si, Cr, W, and Nb atoms, the B-B bond changes little, maintaining to show strong B-B covalent bonding, as listed in Table 5. However, it is worth noting that Si addition contributes to intensifying the Al/Si-B and Al/Si-Mo bonds, by increasing the bond population but decreasing the bond length. As shown in Figure 6b and c, it can be visible that the electron densities between Si-B and Si-Mo are much higher compared with the initial MoAlB crystal. Additionally, the bond length of Si-Al is observed to be shortened due to the smaller radius of Si. Consequently, as a sacrifice, the Mo-B bonds adjacent to the doped Si atom seem to be weaken, as shown in Figure 6c, which can also be confirmed from the decreased population. From Figure 6d, it can be found that the Al-Al bonds can be enhanced to some degree, while the other bonds change little after Cr doping. Intriguingly, W-B and W-Al bonds can be apparently intensified with a higher electron density, as displayed in Figure 6e, which is also consistent with the results of bond population in Table 5. Thus, the improvement of the elastic properties of MoAlB after W doping can be explained from the perspective of bonding characters. For Mo_3_NbAl_4_B_4_, nearly all bonds can be extended due to the larger atomic radius, so that the bonding strength around Nb can be weakened somewhat. The worst mechanical performances of Mo_3_NbAl_4_B_4_ are reasonable from the discussion above.

### 3.5. Debye Temperature

Debye temperature (*θ_D_*) is an important fundamental parameter in evaluating the physical properties of solids, for instance, the melting temperature, thermal expansion, and specific heat. It can be estimated by the average sound velocity (*v_m_*), according to Equation (20) [49,50,60,61]:

(20) $$\theta_{D}=\frac{h}{k_{B}}{\left[\frac{3n}{4\pi }\left(\frac{N_{A}\rho }{M}\right)\right]}^{1/3}v_{m}$$

where *k_B_*, *h*, and *N_A_* are the Boltzmann, Planck, and Avogadro constants, respectively; *n* is the number of atoms in each formula; *ρ* is the density; and *M* is the molecular weight. Herein, the average sound velocity (*v_m_*) can be calculated by Equation (21) [49,50,60]:

(21) $$v_{m}={\left[\frac{1}{3}\left(\frac{2}{v_{t}^{3}}+\frac{1}{v_{l}^{3}}\right)\right]}^{-1/3}$$

where *v_t_* and *v_l_* are the transversal and longitudinal sound velocities, respectively. The *v_t_* and *v_l_* can be obtained by the elastic moduli *B* and G, according to Navier’s equation [49,50,60], as shown below:

(22) $$v_{t}=\sqrt{\frac{G}{\rho }}$$

(23) $$v_{l}=\sqrt{\frac{B+\frac{4}{3}G}{\rho }}$$

**Figure 7:**
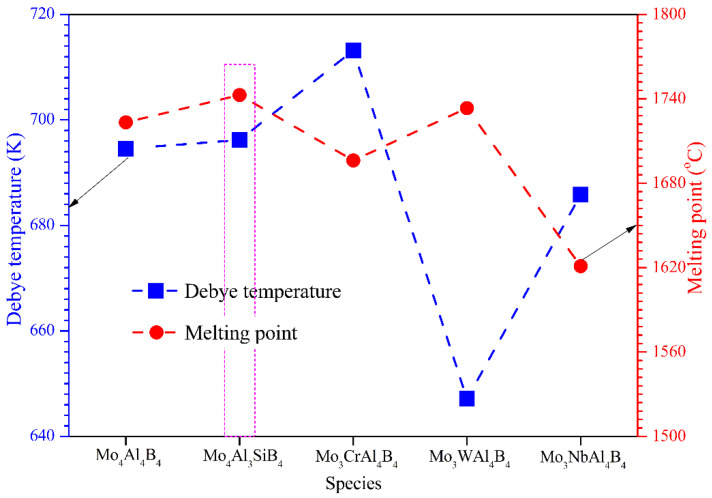
Debye temperature and melting point.

The calculated sound velocities and Debye temperatures (*θ_D_*) of the five calculated crystals have been summarized in Table 6. The Debye temperature of MoAlB is considerably high, indicating its correspondingly good thermal conductivity and strong average chemical bonding. After the introduction of Si, Cr, W, and Mo atoms, *V_t_* and *V_l_* change little, except that the *V_l_* of Mo_3_WAl_4_B_4_ decreases dramatically. Not surprisingly, the average sound velocity shows a remarkable decrease, accounting for the lowest *θ_D_* of Mo_3_WAl_4_B_4_, as shown in Figure 7. In this case, W doping is supposed to weaken the average atomic bonding of MoAlB. By comparison, Si and Cr doping can trigger the increase of *θ_D_* of MoAlB. On the other hand, the melting temperature (*T_m_*) can be empirically obtained with the aid of calculated elastic constants by Equation (24) [62]:

(24) $$T_{m}=354+1.5(2C_{11}+C_{33})$$

where *C*_11_ and *C*_33_ are the elastic constants in Table 2, respectively. As the structural material applied in the high-temperature fields, the melting point is a fairly important parameter that affects MoAlB’s application potential. From Figure 7, it can be visibly found that Mo_4_Al_3_SiB_4_ and Mo_3_WAl_4_B_4_ possess higher *T_m_* than the initial MoAlB. Gene.rally, the melting temperature is tightly associated with the strong covalent bonding in the solid. Thus, the considerable *T_m_* can provide some evidence of the enhancement of covalent bonding after Si and W doping. In conclusion, we can conclude that Si is supposed to be the best doping element to ameliorate the mechanical and physical performance of MoAlB, which will be verified through experimental research in the future.

## 4. Conclusions

In the present work, the effects of Si, Cr, W, and Nb doping on the phase stability, mechanical properties, chemical bonding, and electronic structures of MoAlB crystal have been investigated by DFT calculations. The main conclusions can be summarized:

1. The initial MoAlB, as well as the element (Si, Cr, W and Nb) doped crystals, are thermodynamically stable, confirmed by the negative cohesive energy and formation enthalpy.
2. MoAlB possesses a considerably high Young’s modulus, but it is intrinsically brittle with a B/G ratio less than 1.75. Si and W doping can not only enhance the Young’s modulus of MoAlB but also improve its ductility. However, Cr and Nb play a negative role in the elastic moduli as well as the ductility.
3. MoAlB exhibits significant elastic anisotropy: it shows the largest stiffness along the [1 1 1] direction, while it is the most compressible along [0 1 0]. Si and W doping benefit weakening of the anisotropy of Young’s modulus by strengthening the Young’s moduli along [0 1 0].
4. According to the investigation of DOS, Mulliken populations, and electron density distribution, strong covalent bonding is supposed to exist in MoAlB, combined with ionic and metallic bonding. Si doping significantly intensifies the covalent bondings of Si-B, Si-Mo, and B-B, which accounts for the improved mechanical properties.
5. Si doping can simultaneously increase the Debye temperature and melting point of MoAlB, which apparently shows superior ameliorating effects compared to the other elements. Overall, Si is supposed to be the best dopant in optimizing the comprehensive performance of MoAlB.

## Figures and Tables

**Table 1:** The calculated lattice parameters, density, cohesive energy, and formation enthalpy.

Cell Formula	Lattice Parameters (Å)			Vol (Å^3^)	*ρ* (g/cm^3^)	*E_coh_* (eV/atom)	∆*H_r_* (eV/atom)
	*a*	*b*	*c*				
Mo_4_Al_4_B_4_	3.214	14.038	3.110	140.292	6.332	−7.628	−0.449
	3.210 [5]	13.980 [5]	3.100 [5]	─	─	─	─
	3.200 [46]	13.960 [46]	3.100 [46]	─	─	─	─
	3.216 [47]	14.062 [47]	3.103 [47]	─	─	─	─
Mo_4_Al_3_SiB_4_	3.211	13.766	3.122	138.016	6.449	−7.746	−0.426
Mo_3_CrAl_4_B_4_	3.172	13.977	3.080	136.586	5.969	−7.503	−0.416
Mo_3_WAl_4_B_4_	3.216	14.024	3.112	140.334	7.370	−7.633	−0.412
Mo_3_NbAl_4_B_4_	3.226	14.194	3.129	143.283	6.164	−7.590	−0.477

**Table 2:** Elastic constants of MoAlB crystals with, and without, doping (GPa).

Species	*C*_11_	*C*_12_	*C*_13_	*C*_22_	*C*_23_	*C*_33_	*C*_44_	*C*_55_	*C*_66_
Mo_4_Al_4_B_4_	348.66	140.80	150.45	322.30	121.32	398.32	193.60	159.73	176.43
Mo_4_Al_3_SiB_4_	357.48	150.87	149.71	351.81	134.92	393.61	191.41	162.74	176.75
Mo_3_CrAl_4_B_4_	341.88	139.62	139.42	304.52	124.98	393.80	190.72	157.27	173.26
Mo_3_WAl_4_B_4_	350.73	143.95	154.20	333.69	124.09	400.90	194.74	162.60	177.49
Mo_3_NbAl_4_B_4_	326.66	138.54	145.38	297.68	125.97	374.18	185.88	162.37	186.67

**Table 3:** The elastic moduli (GPa), Poisson’s ratios, and B/G ratios (dimensionless).

Species	*B_V_*	*B_R_*	*B_VRH_*	*G_V_*	*G_R_*	*G_VRH_*	*E*	*ν*	*B/G*
Mo_4_Al_4_B_4_	210.49	208.67	209.58	149.73	140.04	144.89	353.26	0.219	1.446
			232.90 [56]			151.20 [56]	372.90 [56]		
Mo_4_Al_3_SiB_4_	219.32	218.94	219.13	150.67	142.18	146.42	359.25	0.227	1.497
Mo_3_CrAl_4_B_4_	205.36	203.29	204.33	146.66	136.31	141.49	344.86	0.219	1.444
Mo_3_WAl_4_B_4_	214.42	212.99	213.71	151.17	141.54	146.35	357.46	0.221	1.460
Mo_3_NbAl_4_B_4_	202.03	200.13	201.08	146.23	132.79	139.51	339.91	0.218	1.441

**Table 4:** The calculated anisotropic indexes of MoAlB crystals (dimensionless).

Species	*A*_1_	*A*_2_	*A*_3_	*A^U^*	*A_B_*	*A_G_*
Mo_4_Al_4_B_4_	1.736	1.337	1.813	0.355	0.435%	3.344%
Mo_4_Al_3_SiB_4_	1.695	1.369	1.735	0.301	0.089%	2.901%
Mo_3_CrAl_4_B_4_	1.670	1.403	1.887	0.390	0.505%	3.658%
Mo_3_WAl_4_B_4_	1.757	1.337	1.790	0.347	0.334%	3.291%
Mo_3_NbAl_4_B_4_	1.813	1.547	2.150	0.516	0.474%	4.817%

**Table 5:** The calculated bond lengths and overlapped populations (*P* (dimensionless) and *L* (Å) representing the population and bond length, respectively).

**Mo_4_Al_4_B_4_**			**Mo_4_Al_3_SiB_4_**			**Mo_3_CrAl_4_B_4_**		
Bonds	*P*	*L* (Å)	Bonds	*P*	*L* (Å)	Bonds	*P*	*L* (Å)
B-B	1.37	1.81	B-B	1.42	1.80	B-B	1.40	1.80
Al-B	0.16	2.32	Al/Si-B	0.20	2.29	Al-B	0.15	2.30
Mo-B	−0.01	2.35	Mo-B	0.00	2.34	Mo/Cr-B	−0.02	2.31
Mo-B	0.66	2.37	Mo-B	0.57	2.38	Mo/Cr-B	0.66	2.34
Al-Al	0.95	2.67	Al/Si-Al	0.95	2.64	Al-Al	0.97	2.66
Al-Mo	0.73	2.71	Al/Si-Mo	0.81	2.69	Al-Mo/Cr	0.71	2.69
Al-Mo	−0.22	2.99	Al-Mo	−0.23	2.87	Al-Cr	−0.18	2.94
Mo-Mo	−0.96	2.94	Mo-Mo	−0.93	2.97	Mo/Cr-Mo	−0.96	2.90
**Mo_3_WAl_4_B_4_**			**Mo_3_NbAl_4_B_4_**					
Bonds	*P*	*L* (Å)	Bonds	*P*	*L* (Å)			
B	1.33	1.82	B-B	1.39	1.82			
Al-B	0.13	2.32	Al-B	0.15	2.36			
Mo/W-B	0.00	2.35	Mo/Nb-B	0.00	2.35			
Mo/W-B	0.72	2.37	Mo/Nb-B	0.65	2.38			
Al-Al	0.92	2.66	Al-Al	0.96	2.68			
Al-Mo/W	0.79	2.71	Al-Mo/Nb	0.73	2.74			
Al-Mo/W	−0.20	2.98	Al-Mo	−0.22	2.98			
Mo-Mo/W	−0.81	2.94	Mo/Nb-Mo	−1.01	2.95			

**Table 6:** The calculated average, transversal, and longitudinal sound velocities of MoAlB.

Species	*V_l_* (Km/s)	*V_t_* (Km/s)	*V_m_*(Km/s)	*θ_D_* (K)
Mo_4_Al_4_B_4_	7.98	4.78	5.29	694.54
Mo_4_Al_3_SiB_4_	8.02	4.77	5.28	696.19
Mo_3_CrAl_4_B_4_	8.11	4.87	5.39	713.18
Mo_3_WAl_4_B_4_	7.45	4.46	4.93	647.12
Mo_3_NbAl_4_B_4_	7.92	4.76	5.26	685.81
